# Moderators of Cognitive Behavioral Treatment for Insomnia on Depression and Anxiety Outcomes

**DOI:** 10.1007/s11920-022-01326-3

**Published:** 2022-01-21

**Authors:** Riya Mirchandaney, Raul Barete, Lauren D. Asarnow

**Affiliations:** Department of Psychiatry and Behavioral Sciences, University of California, San Francisco, 401 Parnassus Avenue, San Francisco, CA 94143 USA

**Keywords:** Insomnia, Cognitive behavioral therapy for insomnia, Major depressive disorder, Generalized anxiety disorder, Posttraumatic stress disorder, Evening preference

## Abstract

**Purpose of Review:**

With a focus on reviewing adequately powered randomized controlled trials, we present recent research on the potential of cognitive behavioral therapy for insomnia (CBT-I) to improve depression and anxiety outcomes among patients with insomnia and one of the following comorbid psychiatric disorders: major depressive disorder (MDD), generalized anxiety disorder (GAD), or posttraumatic stress disorder (PTSD). We also examine potential moderators of CBT-I on depression and anxiety outcomes in this population.

**Recent Findings:**

Despite high comorbidity rates, current behavioral and pharmacological treatments for MDD, GAD, and PTSD do not substantially target or improve insomnia symptoms; residual insomnia is exceedingly common even among patients who experience remission. Insomnia plays a critical role in the onset and maintenance of depression and anxiety, and treating insomnia with CBT-I may improve global outcomes for patients with MDD, GAD, and PTSD.

**Summary:**

CBT-I is superior to traditional depression/anxiety treatment in improving insomnia symptoms among patients with comorbid psychiatric disorders. Results are mixed on whether CBT-I (either alone or augmented with depression/anxiety treatment) is effective in improving overall MDD, GAD, and PTSD outcomes. Evening circadian preference and depression/anxiety symptom severity may moderate the effect of CBT-I on depression and anxiety outcomes.

**Supplementary Information:**

The online version contains supplementary material available at 10.1007/s11920-022-01326-3.

## Introduction

Insomnia is a common clinical condition with severe health consequences—prevalence rates range from 10 to 20% of the adult population for insomnia disorder, and 35 to 50% for insomnia symptoms [1–3]. Insomnia disorder, as defined by the DSM-5, is characterized by a dissatisfaction with sleep quality or quantity due to difficulty initiating and/or maintaining sleep, resulting in clinically significant distress or impairment in functioning [4]. However, insomnia symptoms are listed in the diagnostic criteria for many other psychiatric disorders, and estimates suggest that individuals with insomnia are five times as likely to present with anxiety or depression compared to individuals without insomnia [4, 5].

Cognitive behavioral therapy for insomnia is the first-line treatment for insomnia, recommended before pharmacotherapy—it is the most widely used and widely studied non-drug treatment for insomnia [1]. A large body of research has supported the efficacy of CBT-I in treating chronic insomnia in adults without comorbidities [6]; additionally, recent studies have indicated that CBT-I may also effectively treat insomnia in children and adolescents, pregnant people, older adults, and individuals with comorbid psychiatric and medical conditions [7–9]. Drawing directly from basic science on sleep and circadian rhythms, CBT-I combines multiple treatment elements including sleep education, stimulus control techniques, sleep restriction techniques, and cognitive therapy [1].

Given the high comorbidity rates of insomnia with depression and anxiety, and considering the various limitations of current evidence-based treatments for depression and anxiety, there has been an increase in research on utilizing CBT-I to improve depression and anxiety outcomes in comorbid samples. Focusing on reviewing randomized controlled trials, we present the literature on CBT-I for patients with insomnia as well one of the following comorbid psychiatric disorders: major depressive disorder (MDD), generalized anxiety disorder (GAD), or posttraumatic stress disorder (PTSD). Finally, in order to better understand why some—but not all—patients experience improvements in depression and anxiety symptoms following CBT-I, we highlight recent research on moderators of treatment outcomes among comorbid samples and suggest promising avenues for future research.

## Major Depressive Disorder

Sleep disturbances are reported among up to 90% of patients with major depressive disorder (MDD) [10]. There is evidence for a bidirectional association between insomnia and MDD, although prior research has primarily focused on insomnia as a risk factor for later depression. Among those with and without MDD, sleep disturbance (in particular, difficulty initiating sleep) is one of the more important predictors of a future depressive episode [11, 12••]; conversely, depressive symptoms increase risk for future insomnia [13–15]. The comorbidity between insomnia and MDD is associated with poorer outcomes for both conditions—symptoms of insomnia are associated with greater depressive symptom severity [16], lower rates of remission from depression [17], and higher risk of depression relapse [11]. Insomnia may also be an independent risk factor for suicide, suicidal thoughts and behaviors, and non-suicidal self-injury among both adults and youth [18–21].

Current therapies for MDD are far from faultless—pharmacotherapy often brings side effects as well as withdrawal symptoms when discontinued, psychotherapy is frequently inaccessible, and increased attention is being paid to the problem of treatment-resistant depression [22]. Even with gold-standard antidepressant treatment, a significant portion of patients do not experience remission, and many who do experience remission subsequently relapse—in the STAR*D trial, only 28% of patients experienced remission after 14 weeks of citalopram, and 70% experienced remission after 1 year of additional trials and treatment augmentations [23]. Furthermore, patients with MDD who do experience remission after treatment still report at least one residual depressive symptom, with sleep disturbance being the most common; a few studies even report worsened sleep after antidepressant treatment [24]. Among patients with comorbid insomnia and MDD, CBT-I has been shown to be effective in treating insomnia symptoms and improving sleep [25••, 26]. Taken together, the addition of CBT-I to augment depression outcomes among patients that suffer from both conditions has been researched as an effective behavioral option targeting the unique insomnia-depression connection.

There have been few adequately powered randomized trials conducted among patients with MDD and insomnia which examined the effect of augmenting depression treatment with CBT-I. In the TRIAD study, 150 participants with MDD and insomnia were randomized to one of two treatment arms—both received depression pharmacotherapy in addition to either individual in-person CBT-I or a control therapy for insomnia (quasi-desensitization) [27]. While CBT-I reduced insomnia severity compared to the control group, there were no significant differences in depression outcomes between the groups [27]. In a similar RCT, Carney et al. (2017) randomized 107 adult participants with MDD and insomnia to one of three treatment arms—antidepressant + 4-session CBT-I, CBT-I + placebo pill, or antidepressant + sleep hygiene control [25••]. Although all three groups improved with respect to subjective insomnia measures, only the two CBT-I groups improved on objective (polysomnography) sleep indices while the antidepressant + sleep hygiene group worsened [25••]. Replicating results from the TRIAD study, Carney et al. (2017) also found no significant differences in depression outcomes between the groups.

While depression treatment augmentation with CBT-I does not significantly improve depression outcomes when compared to an active control condition in adequately powered trials among patients with MDD and insomnia, insomnia improvement does still appear to be an important predictor of depression outcome. Indeed, in the aforementioned TRIAD study, improvements in insomnia early in treatment predicted the trajectory of treatment response and served as a mediator for depression remission following CBT-I [27, 28••]. Further analysis of results from the TRIAD study revealed, through growth mixture modeling, three trajectories of treatment following CBT-I for comorbid depression and insomnia—Partial-Responders, Initial-Responders, and Optimal-Responders [28••]. Reinforcing the findings from Manber et al. [27], participants who had the greatest and most rapid reductions in insomnia severity, sleep effort, and unhelpful beliefs about sleep after baseline were most likely to be characterized as Optimal-Responders (13.5% of the sample) with respect to depression remission, and were likely to maintain minimal depression even at the 2-year follow-up [27, 28••]. Similar findings have also been reported using digital CBT-I (dCBT-I): Henry et al. (2020) analyzed results from two large RCTs of dCBT-I (Sleepio) among participants with insomnia and clinically significant depressive symptoms, finding that improvements in insomnia symptoms at mid-intervention mediated 87% of the effects on depressive symptoms at post-intervention [29••]. Similarly, although Carney et al. (2017) did not investigate insomnia improvement as a mediator of depression treatment outcomes, they did find that lower post-treatment insomnia severity was associated with lower depression severity at post-treatment [25••]. Taken together, these findings indicate that improvement in insomnia symptoms is likely a critical component of depression symptom reduction.

Few trials have directly compared CBT-I alone with depression treatment among patients with insomnia and MDD. By including a CBT-I + placebo pill group, Carney et al. (2017) showed that CBT-I with antidepressant pharmacotherapy was not superior to CBT-I alone in improving either sleep or depression symptoms; by including an antidepressant + sleep hygiene control group, they showed that CBT-I led to similar improvements in depression symptoms and significantly greater improvements on objective sleep measures, compared to antidepressant medication alone [25••]. Instead of using a pharmacotherapy comparator, Blom et al. [30] assigned 43 participants with comorbid insomnia and MDD to internet-delivered CBT for either insomnia or depression, finding that both treatments were equally effective in reducing depression severity. However, CBT-I was more effective in reducing insomnia severity, ultimately indicating superiority of CBT-I—these results persisted at the 3-year follow-up [30, 31].

Given that insomnia symptoms often predict and predate the onset of clinical depression, there has also been increased interest in the possibility of CBT-I as a preventative measure for individuals with subclinical depression. A large RCT by Christensen et al. [32] of a 6-week dCBT-I program (SHUTi) compared to an internet-based control program (HealthWatch) among 1149 participants with insomnia and subclinical depression (a score of between 4 and 20 on the Patient Health Questionnaire-9,not MDD) found that SHUTi significantly reduced depressive symptoms as well as suicidal ideation compared to the control at post-treatment [32]. The effect on depressive symptoms—but not the effect on suicidal ideation—was sustained at both 6-month and 18-month follow-ups [33]. While SHUTi improved depressive symptomatology, Christensen et al. [32] did not find a significant difference in rates of MDD diagnoses between the two groups at follow-up [33]. However, a 2020 trial of dCBT-I (Sleepio) in a sample of 1358 adults with minimal to no depression resulted in a 50% reduction of depression incidence 1 year after treatment, compared to the control condition [34••]. Given the early promise of CBT-I as a preventor of depression in subclinical adult samples, future research should consider CBT-I for preventing depression in high-risk samples, such as pregnant people [8], adolescents, and older adults.

Ultimately, CBT-I as a treatment for comorbid depression and insomnia is promising, and, when compared with CBT for depression or antidepressant medications, may be comparably effective for depressive symptoms and superior for insomnia symptoms. However, it appears that when paired with antidepressant medications, CBT-I may not have a significant additive effect on treatment outcomes—i.e., there may be a “ceiling” effect. However, given that there are only two RCTs of antidepressant medication augmentation with CBT-I, further research is necessary before drawing conclusions. Additionally, as more and more research focuses on preventing, as opposed to treating, mental illness, CBT-I stands out as a potential therapy for decreasing the risk of future MDD—one that can be easily and effectively disseminated via digital health.

## Generalized Anxiety Disorder

The majority of individuals with anxiety disorders report poor sleep, and comorbidity rates with insomnia are estimated to be high, ranging from 45 to 70% [35–37]. Not all anxiety disorders are equally comorbid with insomnia—those with a diagnosis of generalized anxiety disorder (GAD), compared to other anxiety disorders, are 140% more likely to also have sleep disturbance [38]. This comorbidity is particularly worrisome, as the presence of poor sleep in addition to an anxiety disorder is associated with decreased health-related quality of life and increased disability [39].

Longitudinal data indicates that anxiety disorders are a risk factor for later insomnia, but not necessarily the other way around [40]. A potential mechanism underlying the anxiety-insomnia association is the role of worry, which is a phenomenological feature of GAD and a critical element in the cognitive model of insomnia—namely, inappropriate worry about sleep and sleep-related consequences may lead to arousal, which perpetuates insomnia [4, 41]. This association has been demonstrated using daily process/ecological momentary assessment approaches. In a non-clinical sample, worry experienced on a particular day was found to predict increased sleep disturbance that night [42]; however, in a sample with GAD, there was a bidirectional association between worry and sleep disturbance, suggesting a mutually maintaining relationship between worry and poor sleep among individuals with clinically significant anxiety [43].

Given this connection between worry and sleep, treating insomnia among individuals with GAD may be a fruitful opportunity for overall symptom reduction—however, there is currently no adequately powered randomized controlled trial of CBT-I among patients with comorbid GAD and insomnia. Belleville et al. [44] compared the efficacy of two treatment sequences (CBT-I followed by CBT-GAD vs. CBT-GAD followed by CBT-I) in a sample of 10 women with comorbid GAD and insomnia [44]. Results indicated that participants who first received CBT-GAD had superior outcomes on anxiety and sleep—however, most participants who were initially treated with CBT-GAD also experienced additional improvements in both anxiety and sleep following CBT-I [44]. Jansson-Fröjmark and Jacobson (2021) conducted an open trial of CBT-I for 24 adults with comorbid GAD and insomnia, finding moderate to large effect sizes on insomnia and GAD symptoms, as well as depression, functional impairment, and quality of life [45••]. Compared to Belleville et al. [44], Jansson-Fröjmark and Jacobson (2021) found stronger effects of CBT-I on anxiety symptoms, possibly due to the emphasis on cognitive components in their implementation of the treatment [44, 45••]. Notably, only 26.1% of the patients reached insomnia remission at follow-up, a rate much lower than prior studies among patients with insomnia only (~ 56%), suggesting that clinically significant anxiety symptoms might hinder the efficacy of CBT-I [45••]. While there may be some promise in CBT-I as a treatment for GAD, neither of the two trials described above were adequately powered or included a control condition—therefore, any conclusions but be considered with caution.

To our knowledge, the two studies with adults described above are the only trials of CBT-I specifically for patients with comorbid GAD and insomnia. A few open trials have assessed the efficacy of CBT-I (often digital CBT-I) in improving anxiety and insomnia symptoms among adolescents from community samples, with mixed results. In the Aslund et al. (2020) open pilot of individual in-person CBT-I among 23 adolescents, anxiety symptoms significantly improved after treatment, but the post-treatment mean anxiety score was above the clinical cutoff [46]. In the Cliffe et al. (2020) open trial of digital CBT-I among 39 adolescents seeking mental health services, there was a post-treatment reduction in anxiety symptoms (with 51% of the sample having clinically elevated symptoms of anxiety at baseline) [47].

Overall, evidence indicates that CBT-I is safe at likely effective in improving insomnia symptoms among patients with GAD, and there is some indication that improvement in anxiety symptoms prior to conducting CBT-I may improve outcomes for both insomnia and anxiety. However, any conclusions about the efficacy of CBT-I in treating comorbid GAD and insomnia must be considered with caution—while there is empirical support for the link between anxiety and sleep, further research is needed to determine if CBT-I can effectively treat both conditions in this population.

## Posttraumatic Stress Disorder

Trauma-exposed populations report higher than average rates of disturbed sleep; in a national sample of 2647 trauma-exposed adults, more than 92% of individuals who met DSM-5 criteria for posttraumatic stress disorder reported at least one sleep disturbance—in fact, sleep disturbance is often considered a “hallmark” feature of PTSD [48]. While comorbidity estimates of insomnia are high, ranging from 39.6 to 81.6%, particular attention must also be paid to nightmares, as well as other parasomnias (e.g., sleep paralysis, sleep talking, hypnogogic hallucinations), which are also highly prevalent among individuals with PTSD [48, 49]. The high comorbidity rates are likely in part due to the fact that sleep disturbances—both insomnia and nightmares—are included as diagnostic criteria for PTSD [4]. However, multiple prospective studies have shown that sleep disturbance often precedes and predicts subsequent PTSD—data from the Millennium Cohort Study indicated that military personnel reporting insomnia symptoms or short sleep duration (< 6 h) prior to deployment had significantly higher odds of developing PTSD after deployment, and these results have been replicated among multiple longitudinal cohorts of military personnel [50–52].

PTSD treatment may not effectively target sleep-related symptoms. A recent RCT of Prolonged Exposure (PE) and Present Centered Therapy (PCT) for active-duty US Army Soldiers with PTSD found that the majority reported clinically significant insomnia even at post-treatment, and 38% still reported nightmares [53••]. While PTSD remitters improved more than non-remitters on sleep-related symptoms, rates of insomnia and nightmares remained significantly higher than the general population, replicating prior findings and indicating the need for a treatment approach for PTSD targeting sleep-specific symptoms [53••, 54].

To our knowledge, there have been only four randomized controlled trials of CBT-I for patients with comorbid PTSD and insomnia [55–57, 58••]. Talbot et al. [55] randomized 45 adults with PTSD and insomnia to either in-person CBT-I or a waitlist control, finding that CBT-I significantly improved both subjective (sleep diary) and objective (polysomnography) sleep outcomes, compared to the waitlist control [55]. However, Talbot et al. [55] failed to find any significant difference in non-sleep PTSD symptoms at post-treatment, suggesting that improving sleep alone may not necessarily improve global PTSD symptoms [55]. Given the role of nightmares in PTSD, three out of the four RCTs mentioned above assessed combining CBT-I with Imagery Rehearsal Therapy (IRT), a treatment which involves rescripting nightmares to reduce their frequency and intensity [56, 57, 58••]. In two separate trials, Margolies et al. [57] and Ulmer et al. [56] both randomized combat veterans with comorbid PTSD and insomnia to either a combination treatment of CBT-I with adjunctive IRT or an inactive control condition [56, 57]. In both trials, individuals in the treatment condition showed significant improvements in both sleep measures and PTSD symptom severity—however, neither trial included a CBT-I only condition [56, 57]. In order to disentangle the effects of CBT-I and IRT, Harb et al. [58••] randomized 108 veterans with PTSD, nightmares, and poor sleep to either CBT-I + IRT or CBT-I alone, finding that both groups experienced reductions in nightmare frequency and improved sleep quality, and CBT-I + IRT was not superior to CBT-I alone [58••]. Notably, participants in this sample were not required to meet criteria for insomnia, but rather recurrent nightmares, highlighting the heterogeneity of PTSD-comorbid sleep problems that may be treatable with CBT-I [58••].

Few studies have assessed mediators and moderators of CBT-I among samples with comorbid PTSD. Kanady et al. [59] focused on the role of fear of sleep, which is a common feature of PTSD and appears to be positively associated with PTSD symptom severity, possibly fueled by the frequency and intensity of nightmares in this population [59]. Using the sample described above in Talbot et al. [55]—i.e., 45 adults with insomnia and PTSD randomized to either 8 weeks of CBT-I or a waitlist control—Kanady et al. [59] found that, consistent with prior research, greater fear of sleep at baseline was associated with greater global PTSD symptom severity [59]. At post-treatment, participants assigned to CBT-I reported decreased fear of sleep compared to the control, and while the authors did not conduct a mediation analysis, a reduction in fear of sleep following CBT-I predicted a reduction in PTSD symptom severity and hypervigilance intensity [59]. Fear of sleep may be a unique mechanism to target when treating patients with comorbid insomnia and PTSD.

Overall, CBT-I shows some promise in treating comorbid insomnia and PTSD, as well as in treating the heterogeneous sleep disturbances associated with PTSD. However, the majority of studies have focused primarily on adult veterans and military samples—in order to improve the generalizability of these results, additional research is needed to assess the efficacy of CBT-I in treating comorbid insomnia and PTSD in children and adolescents, who have unique sleep needs and challenges, as well as adults with varied trauma exposures (e.g., sexual assault, unexpected death, interpersonal violence, and natural disasters) for whom the connection between sleep disturbances and PTSD symptoms may manifest differently.

## Recent Research on Moderators of CBT-I Outcomes with Comorbid Depression and/or Anxiety

### Eveningness Circadian Preference and Depression

Out of all factors assessed for moderation of CBT-I outcomes with comorbid depression or anxiety, evening circadian preference, defined as the behavioral predilection for later sleep and wake timing, has the most robust support. In Bei et al. [60], a heterogeneous sample of 419 adult patients with insomnia participated in group CBT-I [60]. Participants with stronger eveningness preference, as assessed by the Composite Scale of Morningness (CSM), had higher depressive symptom severity at baseline [60], consistent with previous findings that evening circadian preference (ECP) is cross-sectionally and longitudinally associated with MDD diagnosis, increased depression severity, and nonremission of depression [17, 61, 62]. While baseline symptom severity significantly reduced from pre- to post-treatment across the whole sample, those with evening preference experienced less reduction in depressive symptom severity following CBT-I compared to those with intermediate or morning preference.

These results are similar for pharmacotherapy treatment of insomnia—Rumble et al. [63] examined the association between insomnia, chronotype, and suicidal ideation in a sample of 108 participants with comorbid depression and insomnia being treated with SSRIs and randomized to either zolpidem or a placebo control [63]. Regardless of depression severity, insomnia severity, or treatment assignment, eveningness at baseline was independently associated with greater suicidal ideation over the course of treatment, establishing eveningness as a risk factor for suicidality among individuals with comorbid depression and insomnia [63].

The TRIAD study, described earlier, randomized 139 adult participants with comorbid MDD and insomnia disorder to either CBT-I or a control therapy—both groups also received antidepressant medication [64••]. Although eveningness was not associated with depression severity at baseline, greater evening preference was associated with a smaller reduction in depression severity across both treatment groups [64••]. However, a significant group by chronotype interaction was observed such that participants with evening preference experienced improved depression symptoms when they were treated with CBT-I, suggesting that CBT-I may buffer against the risk of poor antidepressant outcome among those with greater evening preference [64••]. The findings underscore the importance of evaluating circadian preference and suggest that treating comorbid depression and insomnia with antidepressant medications could be enhanced by augmenting antidepressant medications with CBT-I for those identified as having a more evening circadian preference.

Collectively, it appears that patients with ECP and MDD are an important, yet underserved, vulnerable sub-group to target for sleep interventions in order to reduce depression and suicide risk. Although individuals with greater eveningness may experience weaker antidepressant effects from both antidepressant medications and CBT-I, augmenting depression treatment with CBT-I may lead to improved outcomes than either treatment alone.

### High Depression and Anxiety Severity

Among samples with insomnia and comorbid depression or anxiety, there is mixed evidence for symptom severity as a moderator of CBT-I outcomes. Henry et al. (2021), conducting a sub-analysis of two large randomized controlled trials of CBT-I for patients with comorbid insomnia and depression, failed to find any significant moderating effect—i.e., CBT-I was equally effective in improving both insomnia and depressive symptoms for patients regardless of baseline depression symptom severity [29••]. Similarly, in a randomized controlled trial, Christensen et al. [32] found an online CBT-I program (SHUTi) to have consistent depression effects among participants with few vs. elevated depression symptoms at baseline [32]. The above findings, however, are not consistent with results from the Sandlund and Norell-Clarke [65] RCT of CBT-I for primary care patients with insomnia disorder [65]. In this sample, baseline depressive symptoms were a significant predictor of remission, while baseline insomnia symptoms were not—i.e., participants with greater baseline depressive symptom severity were less likely to experience insomnia remission after treatment [65].

To our knowledge, only one study has assessed depression or anxiety symptom severity as a moderator of CBT-I outcomes among adolescents, with somewhat counterintuitive findings. In an RCT of a sleep intervention (“Sleep SENSE,” which included cognitive behavioral and mindfulness components) for 123 adolescents with sleep problems and elevated anxiety symptoms, adolescents with higher baseline anxiety and depression symptoms experienced significant improvements in subjective sleep quality compared to those with lower internalizing symptoms at baseline [66]. This effect could potentially be attributed to the specificities of the Sleep SENSE intervention, which incorporated both sleep- and anxiety-specific modules—in contrast to the “manualized” CBT-I used in the adult trials [66]. However, it is also possible that “at-risk” adolescents may be more motivated or ready for change than those with less severe internalizing problems [66]. In determining who is likely to respond to CBT-I, further research is certainly needed to determine the role of depression and anxiety symptom severity for both adults and adolescents with comorbid insomnia.

## Conclusions

Research on cognitive behavioral therapy for insomnia for comorbid depression and anxiety underscores the importance of treating insomnia symptoms, which are often insufficiently addressed by current depression and anxiety treatments despite being frequently comorbid and mechanistically related to the onset and maintenance of these disorders. The evidence suggests that CBT-I may treat both insomnia and depression/anxiety symptoms in comorbid populations and may even prevent the development of full-fledged MDD, GAD, or PTSD—however, not all patients experience symptom reduction or remission. Future research should investigate evening circadian preference and depression/anxiety symptom severity as potential moderators of treatment outcomes. Future studies will be necessary to determine if CBT-I can also effectively treat comorbid depression and anxiety in children and youth populations.

## Supplementary Information

Below is the link to the electronic supplementary material.

Supplementary file1 (DOCX 13 KB)

Supplementary file2 (PDF 1225 KB)

Supplementary file3 (PDF 1225 KB)

Supplementary file4 (PDF 1225 KB)

## References

[1] Buysse DJ. Insomnia. JAMA. 2013;309(7):706–16. 10.1001/jama.2013.193 10.1001/jama.2013.193 PMC3632369 23423416

[2] Walsh JK, Coulouvrat C, Hajak G, Lakoma MD, Petukhova M, Roth T (2011). Nighttime insomnia symptoms and perceived health in the America Insomnia Survey (AIS). Sleep.

[3] Roth T, Coulouvrat C, Hajak G, Lakoma MD, Sampson NA, Shahly V (2011). Prevalence and perceived health associated with insomnia based on DSM-IV-TR; international statistical classification of diseases and related health problems, tenth revision; and research diagnostic criteria/international classification of sleep disorders, criteria: results from the America insomnia survey. Biol Psychiat.

[4] Association AP. Diagnostic and statistical manual of mental disorders (DSM-5®). American Psychiatric Pub. 2013. 10.1590/s2317-17822013000200017 24413388

[5] Pearson NJ, Johnson LL, Nahin RL (2006). Insomnia, trouble sleeping, and complementary and alternative medicine: analysis of the 2002 national health interview survey data. Arch Intern Med.

[6] Trauer JM, Qian MY, Doyle JS, Rajaratnam SM, Cunnington D (2015). Cognitive behavioral therapy for chronic insomnia: a systematic review and meta-analysis. Ann Intern Med.

[7] Dewald-Kaufmann J, de Bruin E, Michael G (2019). Cognitive behavioral therapy for insomnia (CBT-i) in school-aged children and adolescents. Sleep Med Clin.

[8] Felder JN, Epel ES, Neuhaus J, Krystal AD, Prather AA (2020). Efficacy of digital cognitive behavioral therapy for the treatment of insomnia symptoms among pregnant women: a randomized clinical trial. JAMA Psychiat.

[9] Manber R, Bei B, Simpson N, Asarnow L, Rangel E, Sit A (2019). Cognitive behavioral therapy for prenatal insomnia: a randomized controlled trial. Obstet Gynecol.

[10] Tsuno N, Besset A, Ritchie K (2005). Sleep and depression. J Clin Psychiatry.

[11] Buysse DJ, Angst J, Gamma A, Ajdacic V, Eich D, Rössler W (2008). Prevalence, course, and comorbidity of insomnia and depression in young adults. Sleep.

[12] •• Blanken TF, Borsboom D, Penninx BW, Van Someren EJ. Network outcome analysis identifies difficulty initiating sleep as a primary target for prevention of depression: a 6-year prospective study. Sleep. 2020;43(5):zsz288. **Insomnia severity, specifically sleep duration, predicted onset of MDD in this community sample of 768 adults.** 10.1093/sleep/zsz288 PMC7215262 31789381

[13] Sivertsen B, Salo P, Mykletun A, Hysing M, Pallesen S, Krokstad S (2012). The bidirectional association between depression and insomnia: the HUNT study. Psychosom Med.

[14] Fang H, Tu S, Sheng J, Shao A (2019). Depression in sleep disturbance: a review on a bidirectional relationship, mechanisms and treatment. J Cell Mol Med.

[15] Baglioni C, Battagliese G, Feige B, Spiegelhalder K, Nissen C, Voderholzer U (2011). Insomnia as a predictor of depression: a meta-analytic evaluation of longitudinal epidemiological studies. J Affect Disord.

[16] Alvaro PK, Roberts RM, Harris JK (2013). A systematic review assessing bidirectionality between sleep disturbances, anxiety, and depression. Sleep.

[17] Chan JWY, Lam SP, Li SX, Yu MWM, Chan NY, Zhang J (2014). Eveningness and insomnia: independent risk factors of nonremission in major depressive disorder. Sleep.

[18] Woznica AA, Carney CE, Kuo JR, Moss TG (2015). The insomnia and suicide link: toward an enhanced understanding of this relationship. Sleep Med Rev.

[19] Bernert RA, Kim JS, Iwata NG, Perlis ML (2015). Sleep disturbances as an evidence-based suicide risk factor. Curr Psychiatry Rep.

[20] Kearns JC, Coppersmith DD, Santee AC, Insel C, Pigeon WR, Glenn CR (2020). Sleep problems and suicide risk in youth: a systematic review, developmental framework, and implications for hospital treatment. Gen Hosp Psychiatry.

[21] Latina D, Bauducco S, Tilton‐Weaver L. Insomnia symptoms and non‐suicidal self‐injury in adolescence: understanding temporal relations and mechanisms. J Sleep Res. 2021;30(1):e13190. 10.1111/jsr.13190 PMC7900995 32893426

[22] Pandarakalam JP (2018). Challenges of treatment-resistant depression. Psychiatr Danub.

[23] Insel TR, Wang PS (2009). The STAR* D trial: revealing the need for better treatments. Psychiatr Serv.

[24] Nierenberg A, Husain M, Trivedi M, Fava M, Warden D, Wisniewski S (2010). Residual symptoms after remission of major depressive disorder with citalopram and risk of relapse: a STAR* D report. Psychol Med.

[25] •• Carney CE, Edinger JD, Kuchibhatla M, Lachowski AM, Bogouslavsky O, Krystal AD, et al. Cognitive behavioral insomnia therapy for those with insomnia and depression: a randomized controlled clinical trial. Sleep. 2017;40(4):zsx019. **In this RCT of 107 adults with comorbid insomnia and MDD, CBT-I alone led to depression improvements comparable to that of antidepressant pharmacotherapy and superior improvements in sleep.** 10.1093/sleep/zsx019 PMC5806549 28199710

[26] Feng G, Han M, Li X, Geng L, Miao Y. The clinical effectiveness of cognitive behavioral therapy for patients with insomnia and depression: a systematic review and meta-analysis. Evid Based Complementary Altern Med. 2020;2020. 10.1155/2020/8071821 PMC7378630 32733587

[27] Manber R, Buysse DJ, Edinger J, Krystal A, Luther JF, Wisniewski SR (2016). Efficacy of cognitive-behavioral therapy for insomnia combined with antidepressant pharmacotherapy in patients with comorbid depression and insomnia: a randomized controlled trial. J Clin Psychiatry.

[28] •• Bei B, Asarnow LD, Krystal A, Edinger JD, Buysse DJ, Manber R. Treating insomnia in depression: Insomnia related factors predict long-term depression trajectories. J Consult Clin Psychol. 2018;86(3):282. **Analysis of results from the TRIAD study (RCT of CBT-I for patients with comorbid insomnia and MDD) revealed that participants who had early treatment improvements in insomnia were most likely to experience depression remission.** 10.1037/ccp0000282 PMC5841584 29504795

[29] •• Henry AL, Miller CB, Emsley R, Sheaves B, Freeman D, Luik AI, et al. Insomnia as a mediating therapeutic target for depressive symptoms: a sub‐analysis of participant data from two large randomized controlled trials of a digital sleep intervention. J Sleep Res. 2021;30(1):e13140. **Mid-treatment improvements in insomnia symptoms during digital CBT-I treatment predicted improvements in depressive symptoms at post-treatment.** 10.1111/jsr.13140 PMC8150672 32810921

[30] Blom K, Jernelöv S, Kraepelien M, Bergdahl MO, Jungmarker K, Ankartjärn L (2015). Internet treatment addressing either insomnia or depression, for patients with both diagnoses: a randomized trial. Sleep.

[31] Blom K, Jernelöv S, Rück C, Lindefors N, Kaldo V. Three-year follow-up comparing cognitive behavioral therapy for depression to cognitive behavioral therapy for insomnia, for patients with both diagnoses. Sleep. 2017;40(8). 10.1093/sleep/zsx108 28655183

[32] Christensen H, Batterham PJ, Gosling JA, Ritterband LM, Griffiths KM, Thorndike FP (2016). Effectiveness of an online insomnia program (SHUTi) for prevention of depressive episodes (the GoodNight Study): a randomised controlled trial. The Lancet Psychiatry.

[33] Batterham PJ, Christensen H, Mackinnon AJ, Gosling JA, Thorndike FP, Ritterband LM (2017). Trajectories of change and long-term outcomes in a randomised controlled trial of internet-based insomnia treatment to prevent depression. BJPsych open.

[34] •• Cheng P, Kalmbach DA, Castelan AC, Murugan N, Drake CL. Depression prevention in digital cognitive behavioral therapy for insomnia: Is rumination a mediator? J Affect Disord. 2020;273:434–41. **Among adults with minimal to no depression, digital CBT-I resulted in a reduction in depression incidence at 1-year follow-up compared to the control.** 10.1016/j.jad.2020.03.184 32560938

[35] Soehner AM, Harvey AG (2012). Prevalence and functional consequences of severe insomnia symptoms in mood and anxiety disorders: results from a nationally representative sample. Sleep.

[36] Kim B-S, Jeon HJ, Hong JP, Bae JN, Lee J-Y, Chang SM (2012). DSM-IV psychiatric comorbidity according to symptoms of insomnia: a nationwide sample of Korean adults. Soc Psychiatry Psychiatr Epidemiol.

[37] Monti JM, Monti D (2000). Sleep disturbance in generalized anxiety disorder and its treatment. Sleep Med Rev.

[38] Marcks BA, Weisberg RB, Edelen MO, Keller MB (2010). The relationship between sleep disturbance and the course of anxiety disorders in primary care patients. Psychiatry Res.

[39] Ramsawh HJ, Stein MB, Belik S-L, Jacobi F, Sareen J (2009). Relationship of anxiety disorders, sleep quality, and functional impairment in a community sample. J Psychiatr Res.

[40] Johnson EO, Roth T, Breslau N (2006). The association of insomnia with anxiety disorders and depression: exploration of the direction of risk. J Psychiatr Res.

[41] Harvey AG (2002). A cognitive model of insomnia. Behav Res Ther.

[42] McGowan SK, Behar E, Luhmann M (2016). Examining the relationship between worry and sleep: a daily process approach. Behav Ther.

[43] Thielsch C, Ehring T, Nestler S, Wolters J, Kopei I, Rist F (2015). Metacognitions, worry and sleep in everyday life: studying bidirectional pathways using Ecological Momentary Assessment in GAD patients. J Anxiety Disord.

[44] Belleville G, Ivers H, Bélanger L, Blais FC, Morin CM (2016). Sequential treatment of comorbid insomnia and generalized anxiety disorder. J Clin Psychol.

[45] •• Jansson-Fröjmark M, Jacobson K. Cognitive behavioural therapy for insomnia for patients with co-morbid generalized anxiety disorder: an open trial on clinical outcomes and putative mechanisms. Behav Cogn Psychother. 2021:1–16. **In this open pilot, CBT-I led to improvements anxiety and insomnia symptoms for adults with comorbid insomnia and GAD.** 10.1017/S1352465821000023 33504410

[46] Åslund L, Lekander M, Wicksell RK, Henje E, Jernelöv S (2020). Cognitive-behavioral therapy for insomnia in adolescents with comorbid psychiatric disorders: a clinical pilot study. Clin Child Psychol Psychiatry.

[47] Cliffe B, Croker A, Denne M, Smith J, Stallard P. Digital cognitive behavioral therapy for insomnia for adolescents with mental health problems: feasibility open trial. JMIR Mental Health. 2020;7(3):e14842. 10.2196/14842 PMC7078631 32134720

[48] Milanak ME, Zuromski KL, Cero I, Wilkerson AK, Resnick HS, Kilpatrick DG (2019). Traumatic event exposure, posttraumatic stress disorder, and sleep disturbances in a national sample of US adults. J Trauma Stress.

[49] Ohayon MM, Shapiro CM (2000). Posttraumatic stress disorder in the general population. Compr Psychiatry.

[50] Gehrman P, Seelig AD, Jacobson IG, Boyko EJ, Hooper TI, Gackstetter GD (2013). Predeployment sleep duration and insomnia symptoms as risk factors for new-onset mental health disorders following military deployment. Sleep.

[51] Acheson DT, Kwan B, Maihofer AX, Risbrough VB, Nievergelt CM, Clark JW (2019). Sleep disturbance at pre-deployment is a significant predictor of post-deployment re-experiencing symptoms. Eur J Psychotraumatol.

[52] Wright KM, Britt TW, Bliese PD, Adler AB, Picchioni D, Moore D (2011). Insomnia as predictor versus outcome of PTSD and depression among Iraq combat veterans. J Clin Psychol.

[53] •• Taylor DJ, Pruiksma KE, Hale W, McLean CP, Zandberg LJ, Brown L, et al. Sleep problems in active duty military personnel seeking treatment for posttraumatic stress disorder: presence, change, and impact on outcomes. Sleep. 2020;43(10):zsaa065. **Insomnia and nightmares are common among active-duty soldiers, and PTSD treatment does not improve sleep disturbances.** 10.1093/sleep/zsaa065 32246153

[54] Pruiksma KE, Taylor DJ, Wachen JS, Mintz J, Young-McCaughan S, Peterson AL (2016). Residual sleep disturbances following PTSD treatment in active duty military personnel. Psychol Trauma Theory Res Pract Policy.

[55] Talbot LS, Maguen S, Metzler TJ, Schmitz M, McCaslin SE, Richards A (2014). Cognitive behavioral therapy for insomnia in posttraumatic stress disorder: a randomized controlled trial. Sleep.

[56] Ulmer CS, Edinger JD, Calhoun PS (2011). A multi-component cognitive-behavioral intervention for sleep disturbance in veterans with PTSD: a pilot study. J Clin Sleep Med.

[57] Margolies SO, Rybarczyk B, Vrana SR, Leszczyszyn DJ, Lynch J (2013). Efficacy of a cognitive-behavioral treatment for insomnia and nightmares in Afghanistan and Iraq veterans with PTSD. J Clin Psychol.

[58] •• Harb GC, Cook JM, Phelps AJ, Gehrman PR, Forbes D, Localio R, et al. Randomized controlled trial of imagery rehearsal for posttraumatic nightmares in combat veterans. J Clin Sleep Med. 2019;15(5):757–67. **In this RCT, CBT-I alone improved insomnia and PTSD symptoms among veterans, and augmenting CBT-I with IRT did not result in improved treatment outcomes.** 10.5664/jcsm.7770 PMC6510682 31053215

[59] Kanady JC, Talbot LS, Maguen S, Straus LD, Richards A, Ruoff L (2018). Cognitive behavioral therapy for insomnia reduces fear of sleep in individuals with posttraumatic stress disorder. J Clin Sleep Med.

[60] Bei B, Ong JC, Rajaratnam SM, Manber R (2015). Chronotype and improved sleep efficiency independently predict depressive symptom reduction after group cognitive behavioral therapy for insomnia. J Clin Sleep Med.

[61] Chelminski I, Ferraro FR, Petros TV, Plaud JJ (1999). An analysis of the “eveningness–morningness” dimension in “depressive” college students. J Affect Disord.

[62] Abe T, Inoue Y, Komada Y, Nakamura M, Asaoka S, Kanno M (2011). Relation between morningness–eveningness score and depressive symptoms among patients with delayed sleep phase syndrome. Sleep Med.

[63] Rumble ME, McCall WV, Dickson DA, Krystal AD, Rosenquist PB, Benca RM (2020). An exploratory analysis of the association of circadian rhythm dysregulation and insomnia with suicidal ideation over the course of treatment in individuals with depression, insomnia, and suicidal ideation. J Clin Sleep Med.

[64] •• Asarnow LD, Bei B, Krystal A, Buysse DJ, Thase ME, Edinger JD, et al. Circadian preference as a moderator of depression outcome following cognitive behavioral therapy for insomnia plus antidepressant medications: a report from the TRIAD study. J Clin Sleep Med. 2019;15(4):573–80. **Patients with evening preference had worse outcomes following depression treatment, but they experienced greater improvements in depression following CBT-I.** 10.5664/jcsm.7716 PMC6457514 30952216

[65] Sandlund C, Norell-Clarke A (2020). Is it more about mood than about sleep?: an investigation into depression as amoderator and mediator of remission after CBT-I. Sömn och hälsa.

[66] Blake MJ, Blake LM, Schwartz O, Raniti M, Waloszek JM, Murray G (2018). Who benefits from adolescent sleep interventions? Moderators of treatment efficacy in a randomized controlled trial of a cognitive-behavioral and mindfulness-based group sleep intervention for at-risk adolescents. J Child Psychol Psychiatry.

