# Supplementary material for: Moderators of Cognitive Behavioral Treatment for Insomnia on Depression and Anxiety Outcomes
Source: Curr Psychiatry Rep. 2022 Jan 21;24(2):121–8. doi: 10.1007/s11920-022-01326-3 (PMC8948126; doi:10.1007/s11920-022-01326-3)
Supplement: Supplementary file 1 — Supplementary file1 (DOCX 13 KB) [file 11920_2022_1326_MOESM1_ESM.docx]

Dear Dr. Gehrman,

Thank you very much for the thoughtful feedback on our paper 'Moderators of cognitive behavioral treatment for insomnia on depression and anxiety outcomes'. I hope we have responded to your comments appropriately, but please let me know if there is anything we haven’t addressed. **1. Introduction: In the description of CBT-I please provide 1-2 sentences summarizing the efficacy for insomnia outcomes.**

The authors have added the following sentence to the introduction summarizing the efficacy for insomnia outcomes: *“A large body of research has supported the efficacy of CBT-I in treating chronic insomnia in adults without comorbidities [6]; additionally, recent studies have indicated that CBT-I may also effectively treat insomnia in children and adolescents, pregnant people, older adults, and individuals with comorbid psychiatric and medical conditions [7-9].”* **2. Page 7: in the description of the Christensen et al RCT please state what severity of depressive symptoms was required for entry into the study.**

The authors have specified that the inclusion criteria for the Christensen et al. RCT was a PHQ-9 score between 4 and 20.

**3. Page 13: the acronym ECP is used towards the bottom but never defined.**

The authors have added the definition of ECP (evening circadian preference) upon first mention of the term.

Sincerely,

Riya Mirchandaney
